# Vaccination With a FAT1-Derived B Cell Epitope Combined With Tumor-Specific B and T Cell Epitopes Elicits Additive Protection in Cancer Mouse Models

**DOI:** 10.3389/fonc.2018.00481

**Published:** 2018-10-26

**Authors:** Alberto Grandi, Laura Fantappiè, Carmela Irene, Silvia Valensin, Michele Tomasi, Simone Stupia, Riccardo Corbellari, Elena Caproni, Ilaria Zanella, Samine J. Isaac, Luisa Ganfini, Luca Frattini, Enrico König, Assunta Gagliardi, Simona Tavarini, Chiara Sammicheli, Matteo Parri, Guido Grandi

**Affiliations:** ^1^Toscana Life Sciences, Siena, Italy; ^2^CIBIO, University of Trento, Trento, Italy; ^3^GSK Vaccines, Siena, Italy

**Keywords:** FAT1 cadherin, outer membrane vesicle (OMV), cancer vaccines, personalized medicine, antibody, cell mediated immunity

## Abstract

Human FAT1 is overexpressed on the surface of most colorectal cancers (CRCs) and in particular a 25 amino acid sequence (D8) present in one of the 34 cadherin extracellular repeats carries the epitope recognized by mAb198.3, a monoclonal antibody which partially protects mice from the challenge with human CRC cell lines in xenograft mouse models. Here we present data in immune competent mice demonstrating the potential of the D8-FAT1 epitope as CRC cancer vaccine. We first demonstrated that the mouse homolog of D8-FAT1 (mD8-FAT1) is also expressed on the surface of CT26 and B16F10 murine cell lines. We then engineered bacterial outer membranes vesicles (OMVs) with mD8-FAT1 and we showed that immunization of BALB/c and C57bl6 mice with engineered OMVs elicited anti-mD8-FAT1 antibodies and partially protected mice from the challenge against CT26 and EGFRvIII-B16F10 cell lines, respectively. We also show that when combined with OMVs decorated with the EGFRvIII B cell epitope or with OMVs carrying five tumor-specific CD4+ T cells neoepitopes, mD8-FAT1 OMVs conferred robust protection against tumor challenge in C57bl6 and BALB/c mice, respectively. Considering that FAT1 is overexpressed in both KRAS^+^ and KRAS^−^ CRCs, these data support the development of anti-CRC cancer vaccines in which the D8-FAT1 epitope is used in combination with other CRC-specific antigens, including mutation-derived neoepitopes.

## Introduction

Human FAT atypical cadherin 1 (FAT1) is a type 1 transmembrane protein carrying 34 cadherin repeats, five EGF-like repeats, a laminin A–G domain in the extracellular region and a cytoplasmic tail (1). The protein undergoes a proteolytic cleavage by Furin and is predicted to be further cleaved by γ secretase so that its intracellular domain (ICD) can translocate into the nucleus and directly activate cell signaling. FAT1 ICD also interacts with Ena/VAPS and Scribble, promotes actin-mediated cell migration and inhibits YAP1-mediated cell proliferation (2). In addition, FAT1 ICD also interacts with β-catenin and prevents its translocation to the nucleus (3).

Alteration of FAT1 expression and function has been associated to several human cancers. Although its role in tumorigenesis is still controversial, in some cancers such as acute myeloid leukemia (AML), pre-B acute lymphoblastic leukemia (ALL), T-ALL, and hepatocarcinoma, FAT1 has been described to act as tumor promoter (4, 5). FAT1 up-regulation is an unfavorable prognostic factor for precursor B-cell acute lymphoblastic leukemia patients (4) and recent studies in melanoma and pancreatic cancer have demonstrated that FAT1 undergoes an aberrant processing and an altered localization compared to normal cells (6).

Recently, Pileri et al. (7) discovered that FAT1 is over-expressed in a large fraction of early and late stage colorectal cancers (CRCs). Moreover, a murine monoclonal antibody (mAb198.3), recognizing an epitope present within a 25 amino acid region of the cadherin domain 8 (hereinafter D8-FAT1), was shown to selectively bind the surface of different FAT1-positive CRC cell lines. Moreover, all CRC-derived liver metastases so far tested are highly positive mAb198.3 staining. Interestingly, using extensive immunohistochemistry analysis, the same authors demonstrated that not only mAb198.3 stained 93% of 642 CRC samples tested but also the antibody did not recognize a large panel of human healthy tissues. This strongly suggests that FAT1 can be exploited as a novel target for CRC immunotherapy. Indeed, it was demonstrated that in immunocompromised mice challenged with human colon CRC cells, mAb198.3 accumulated at tumor sites and partially inhibited tumor growth (7).

In our laboratories we have been exploiting bacterial Outer Membrane Vesicles (OMVs) as a vaccine platform (8). OMVs, 20–300 nm closed spheroid particles (9), are particularly attractive for vaccine applications for three main reasons. First, they carry many Microbe-Associated Molecular Patterns (MAMPs), which work synergistically and stimulate potent Th1-skewed immune responses (10–12). Second, OMVs can be easily decorated with foreign antigens/epitopes by properly manipulating the OMV-producing strains (13–17). Third, OMVs can be efficiently purified from bacterial culture supernatants (18, 19). Indeed, OMVs have already been exploited in human vaccination and they represent a key component of the anti-Meningococcus B vaccine (Bexsero) currently available in Europe and the USA.

The recent data demonstrating that vaccines constituted by mutation-derived CD4+/CD8+ T cell neoepitopes induce anti-tumor immune responses in both preclinical and clinical settings (20, 21), had prompted us to test whether the unique properties of OMVs could be exploited in cancer immunotherapy. We already showed that immunization with OMVs engineered with the EGFRvIII tumor-specific B cell epitope (22) and with M30, a mutation-derived CD4+ T cell epitope expressed in B16F10 murine melanoma cells (23), fully prevented tumor growth in C57bl6 mice challenged with B16F10 cells expressing EGRFvIII (24). We also showed that protection was associated to the elicitation of both anti-EGFRvIII antibodies and M30-specific T cells.

In this work we have investigated whether OMVs could be engineered with the D8-FAT1 domain and whether D8-FAT1-decorated OMVs could induce anti-tumor immune responses against FAT1-positive tumors. Here we show that several murine cancer cell lines, including the colon cancer cell line CT26, expose FAT1 on their surface. We also show that OMVs can be efficiently decorated with D8-FAT1 epitope and that D8-FAT1-OMVs induce high levels of anti-FAT1 antibodies. Furthermore, immunization with D8-FAT1-OMVs partially prevents tumor growth in BALB/c mice challenged with CT26 cell line. Finally, we show that when combined with other cancer specific-epitopes, D8-FAT1 provides an additive effect and potentiates the overall anti-tumor immune responses.

Taken together these data strengthen the association of FAT1 expression in CRC and other tumors and pave the way to the use of D8-FAT1 epitope in cancer immunotherapy, particularly in association with other tumor-specific epitopes.

## Results

### FAT1 is expressed in murine cancer cell lines

**Figure 1:**
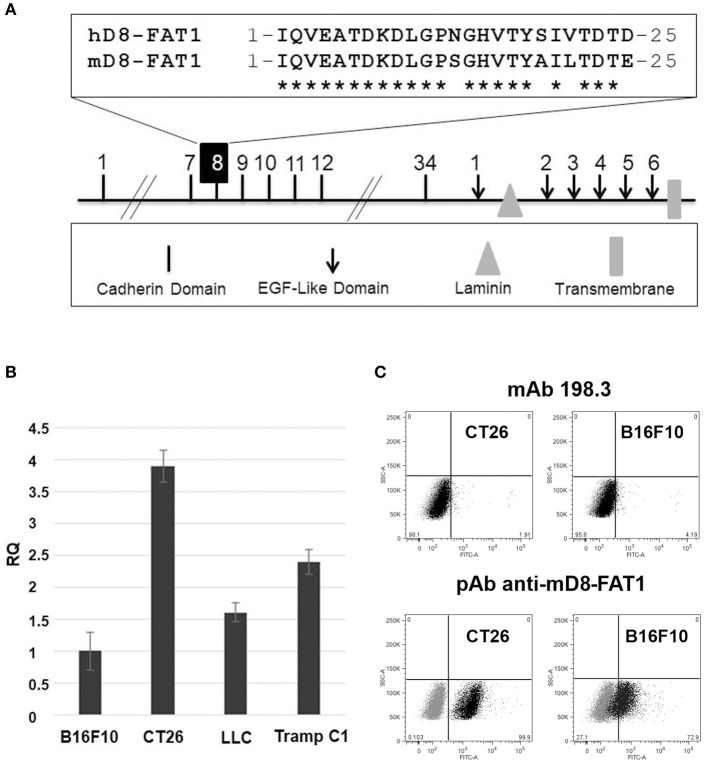
Analysis of FAT1 expression in murine cell lines. **(A)** *Schematic representation of the structural organization of FAT1*. Highlighted is the comparison between the 25 amino acid sequence of the human cadherin domain 8 (hD8-FAT1) recognized by mAb198.3 (7) and the corresponding sequence in murine FAT1 (mD8-FAT1). **(B)** *Quantitative analysis of FAT1 mRNA in mouse cancer cell lines*—mRNA was purified from different cancer cells lines and qRT-PCR was carried out to quantify FAT1-specific mRNA. Data are reported as fold differences with respect to FAT1 mRNA from B16F10 cell line. The bars represent the means ± SD of three independent experiments. **(C)** *Surface exposition of mD8-FAT1 domain in B16F10 and CT26 cell lines*. Cancer cells were incubated with either mAb198.3 monoclonal antibodies or with polyclonal antibodies raised against the KLM-conjugated synthetic peptide corresponding to the mD8-FAT1 **(A)**. Cells were subsequently incubated with fluorescent labeled secondary antibodies and analyzed by flow cytometry.

As already pointed out, FAT1 is over-expressed in most human CRCs and the 25 amino acid hD8-FAT1 domain recognized by mAb198.3 is exposed on cancer cells and not on healthy human tissues. Therefore, hD8-FAT1 represents a novel tumor specific-epitope which could be potentially exploited in immunotherapeutic vaccines. A prerequisite to test this hypothesis in tumor models of immunocompetent mice is that D8-FAT1 is expressed on the surface of syngeneic murine cancer cell lines. Murine FAT1 (mFAT1) shares 88% identity to hFAT1 and in particular 21 out of the 25 amino acids of hD8-FAT1 are conserved in murine D8-FAT1 (mD8-FAT1) (Figure 1A). However, to the best of our knowledge, little was known about FAT1 expression and compartmentalization in mouse cancer cells. To investigate this, we first purified total mRNA from CT26, B16F10, LLC, and Tramp C1 cells and the presence of FAT1-specific transcripts were analyzed by qRT-PCR. As shown in Figure 1B, FAT1-specific mRNA was present in all cancer cells and, interestingly, FAT1 mRNA was particularly abundant in CT26 cell line, a colon cancer cell line derived from BALB/c mice.

We next investigated the presence of the mD8-FAT1 domain on the surface of B16F10 and CT26 cells by flow cytometry analysis using mAb198.3. Neither cell line was recognized by the monoclonal antibody (Figure 1C). This negative result could be attributed either to a difference in cellular surface expression of FAT1 between human and mouse cancer cells or to the inability of mAb198.3 to bind mD8-FAT1 due to the four amino acid difference present in the D8-FAT1 sequences of the two species. To discriminate between the two possibilities, polyclonal antibodies against a synthetic peptide corresponding to the 25 amino acid sequence of mD8-FAT1 were generated in rabbits and the serum was used to detect mD8-FAT1 surface expression by flow cytometry. As shown in Figure 1C, the anti-mD8-FAT1 antibodies specifically bound both B16F10 and CT26 cells, FAT1 surface expression being higher in CT26, in line with the RNA data.

### OMVs can be decorated with mD8-FAT1

**Figure 2:**
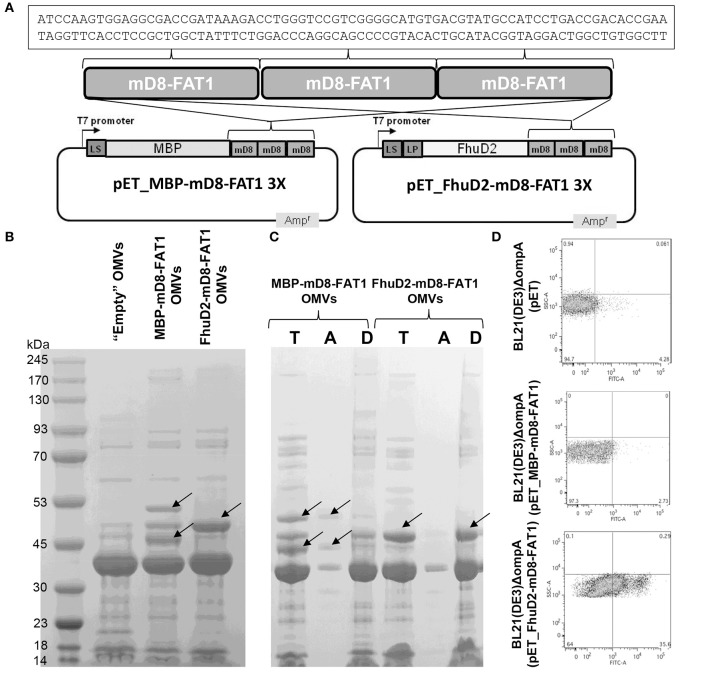
Expression and localization of mD8-FAT1 fusion proteins. **(A)** *Schematic representation of plasmids expressing mD8-FAT1 fusion proteins*. A synthetic minigene encoding three copies of mD8-FAT1 domain was fused to the 3′ end of either *E. coli* Maltose binding protein (MBP) gene or *S. aureus fhuD2* gene. The two fusions were inserted into pET plasmid under the control of the T7 inducible promoter. Highlighted is the DNA sequence of the mD8-FAT1 minigene. **(B)** *Compartmentalization of mD8-FAT1 fusions in OMVs*. OMVs were purified from the supernatants of BL21(DE3)Δ*ompA*(pET_MBP-mD8-FAT1) and BL21(DE3)Δ*ompA*(pET_FhuD2-mD8-FAT1) strains and 20 μg of each OMVs preparation were separated by SDS-PAGE and the gel was stained with Coomassie Blue. As control, OMVs from BL21(DE3)Δ*ompA*(pET) strain (“Empty” OMVs) were also loaded on the gel. Arrows indicate the bands corresponding to the protein fusions. **(C)** *Analysis of compartmentalization of mD8-FAT1 fusions in OMVs by Triton X-114 extraction*. OMVs (100 μg) from BL21(DE3)Δ*ompA*(pET_MBP-mD8-FAT1) and BL21(DE3)Δ*ompA*(pET_FhuD2-mD8-FAT1) strains were incubated in 1% Triton X-114 solution at 4°C and subsequently aqueous and hydrophobic phases were separated by bringing the temperature at 37°C. Proteins in the aqueous **(A)** and hydrophobic **(D)** phases were precipitated by standard chloroform/methanol procedure, separated by SDS-PAGE together with 20 μg of OMVs and stained with Coomassie blue (T). **(D)** *Analysis of surface localization of mD8-FAT1 fusion proteins*. Bacterial cells from BL21(DE3)Δ*ompA*(pET_MBP-mD8-FAT1) and BL21(DE3)Δ*ompA*(pET_FhuD2-mD8-FAT1) cultures were first incubated with anti-mD8-FAT1 polyclonal antibodies and subsequently with fluorescent-labeled anti-rabbit antibodies. Antibody binding was visualized by flow cytometry.

The demonstration of FAT1 expression and D8-FAT1 surface exposition in cancer cell lines prompted us to produce OMVs decorated with mD8-FAT1 with the aim of using mD8-FAT1-decorated OMVs in mouse immunogenicity studies. To load OMVs with mD8-FAT1, a synthetic minigene encoding three copies of the 25 amino acid mD8-FAT1 domain was fused to the 3' end of the genes encoding the *E. coli* periplasmic Maltose Binding Protein (MBP) (25) and the *Staphylococcus aureus* FhuD2 lipoprotein (26) (Figure 2A). The two gene fusions were inserted into pET plasmid under the control of the IPTG-inducible T7 promoter and plasmids pET_MBP-mD8-FAT1 and pET_FhuD2-mD8-FAT1 thus generated were used to transform *E. coli* BL21(DE3)Δ*ompA*, a strain featuring an OMV over-producing phenotype (16). After 2-hour induction of protein expression, OMVs were purified from the culture supernatants and the accumulation of the fusion proteins in the OMVs was analyzed by SDS-PAGE. As shown in Figure 2B, protein bands corresponding to the expected molecular masses of MBP-mD8-FAT1 (51 kDa) and tri-acylated FhuD2-mD8-FAT1 (approx. 45 kDa), were clearly visible on the gel. In the case of MBP-mD8-FAT1-OMVs a second band of ~45 kDa is also visible. The protein is likely to correspond to a degradation product, which is however still recognized by mD8-FAT1 antibodies (data not shown). MBP is a periplasmic protein while FhuD2 is a lipoprotein which is expected to reach the outer membrane. Therefore, C-terminal fusions to MBP and FhuD2 should reside in the luminal and in the membrane compartments of OMVs, respectively. The different compartmentalization of the two fusion proteins in the OMVs was indirectly confirmed by solubilizing the OMVs with 1% Triton X-114 at 4°C and by following the partition of the fusion proteins in aqueous and detergent phases, which separate upon temperature shifting at 37°C. Under these conditions membrane proteins and lipoproteins typically partition into the Triton X-114 hydrophobic phase while periplasmic proteins in the hydrophilic one (17). As shown in Figure 2C, FhuD2-mD8-FAT1 compartmentalized in the detergent phase while the MBP-mD8-FAT1 fusion in the aqueous phase.

The localization of the two fusions was also analyzed by flow cytometry analysis of BL21(DE3)Δ*ompA*(pET_MBP-mD8-FAT1) and BL21(DE3)Δ*ompA*(pET_FhuD2-mD8-FAT1) cells, using mD8-FAT1-specific antibodies. As shown in Figure 2C, while the antibodies bound the cell surface of the strain expressing the FhuD2-mD8-FAT1 fusion, no appreciable florescent shift was observed in the strain expressing the MBP-mD8-FAT1 fusion. These data also indicate that the FhuD2-mD8-FAT1 fusion not only resides in the outer membrane of *E. coli* but also protrudes out of the cell surface, thus making the mD8-FAT1 epitope accessible to antibody binding. This is an interesting observation since *E. coli* does not expose most of its outer membrane lipoproteins and this is often attributed to the absence of specific “flippases” that allow lipoproteins to move from the inner to the outer leaflet of the outer membrane. The fact that FhuD2 lipoprotein is surface-exposed, supports our previous observations that in Gram-negative bacteria many lipoproteins, in the absence of still poorly characterized retention signals, are “by default” destined to cross the outer membrane (17).

### mD8-FAT1-OMVs immunization inhibits tumor growth in CT26-challenged mice

**Figure 3:**
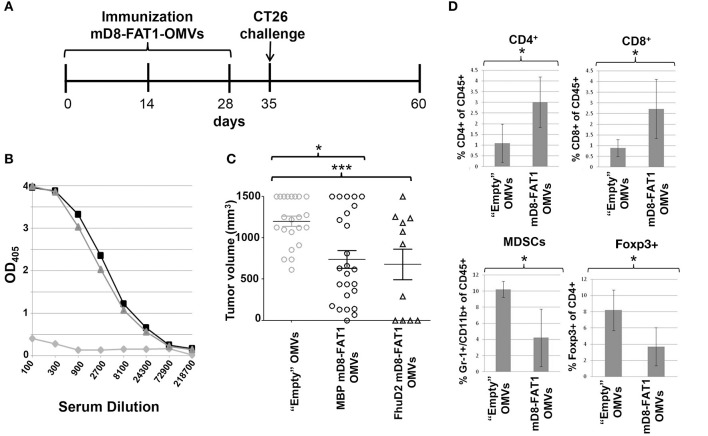
Protection conferred by mD8-FAT1 OMVs immunization against CT26 challenge. **(A)** *Schematic representation of immunization and challenge schedule*. BALB/c mice were immunized three times (2 weeks apart) with OMVs from either BL21(DE3)Δ*ompA*(pET_MBP-mD8-FAT1) or BL21(DE3)Δ*ompA*(pET_FhuD2-mD8-FAT1) strains and 1 week after the third immunization the animals were challenged with 2 × 10^5^ CT26 cells. Tumor growth was followed over a period of 25 days. As control, a group of mice was also immunized with “Empty” OMVs. **(B)** *anti-mD8-FAT1 titers from mice immunized with mD8-FAT1 OMVs*. The day before challenge sera from immunized mice were pooled (triangles: mice immunized with MBP-mD8-FAT1-OMVs; squares: mice immunized with FhuD2-mD8-FAT1-OMVs; circles: mice immunized with “Empty” OMVs) and the anti-mD8-FAT1 titers were determined by ELISA using plates coated with synthetic mD8-FAT1 peptide. **(C)** *Anti-tumor activity of mD8-FAT1 OMVs immunizations*. After challenge tumor growth was followed by measuring tumor volume with a caliper. Animals were sacrificed 25 days after challenge. Means ± SEM are indicated. ***Indicates that the difference in tumor size between the immunized group and control group is statistically significant with *P* < 0.001, while *indicates *P* < 0.05. **(D)** *Analysis of immune cell populations in tumors*. At day 25 from challenge, tumors were collected from sacrificed mice and the frequencies of CD4+ T cells, CD8+ T cells, MDSCs and CD4+/Foxp3+ T cells in tumors were determined by flow cytometry. The data reported in the figure represents the means ± SD of cell populations from four tumors collected from mice immunized with either “Empty OMVs” or with mD8-FAT1 OMVs (two mice from MBP-mD8-FAT1 OMVs and two mice from FhuD2-mD8-FAT1 OMVs) (*P < 0.05).

We next asked the question whether immunization with mD8-FAT1-decorated OMVs could elicit anti-mD8-FAT1 antibodies in mice. To this aim, BALB/c mice were immunized three times (Figure 3A) with either MBP-mD8-FAT1-OMVs (20 μg/dose supplemented with Alum) or with FhuD2-mD8-FAT1-OMVs (20 μg/dose) and 1 week after the third immunization sera from each group were pooled together and analyzed by ELISA using plates coated with the synthetic mD8-FAT1 peptide. As shown in Figure 3B, both immunizations induced high titers of mD8-FAT1 specific antibodies. In line with a previously published work (16), no appreciable difference was observed between titers elicited by OMVs carrying D8-FAT1 on the surface or in the lumen.

Immunized animals were subsequently challenged with CT26 cells and tumor growth was followed over a period of 25 days. Both immunizations inhibited tumor progression in a statistically significant manner, and after 25 days from challenge tumor volumes were ~50% smaller than those measured in mice immunized with “empty” OMVs (Figure 3C). We also analyzed the immune cell population in tumors from control mice and from mice immunized with mD8-FAT1-decorated OMVs. As shown in Figure 3D, tumor inhibition in mice immunized with mD8-FAT1-OMVs was accompanied by the accumulation of infiltrating CD8+ and CD4+ T cells and by the concomitant reduction of regulatory T cells (CD4+/Foxp3+) and myeloid-derived suppressor cells (MDSCs).

### mD8-FAT1-OMVs immunization cooperates with OMVs decorated with other cancer-specific B cell epitopes

Because of the heterogeneity of the cancer cell population and of the immune-editing mechanism that allow cancer cells to escape immune surveillance, to be effective cancer vaccines should be formulated with more than one tumor-specific/associated antigen. Therefore, we first tested whether mD8-FAT1 could be utilized in combination with other B cell epitopes selectively expressed in cancer cells.

**Figure 4:**
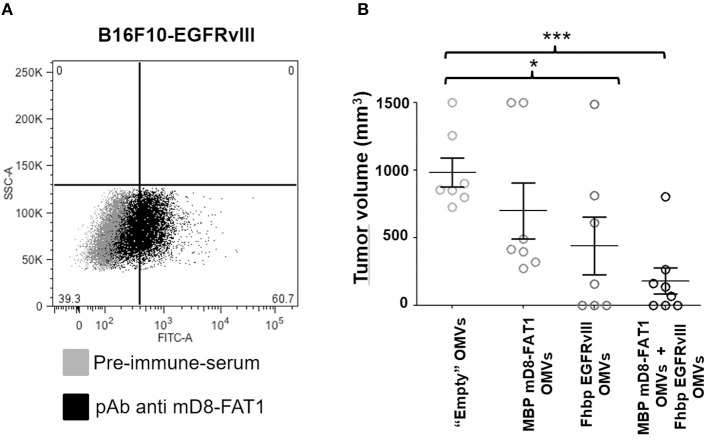
Protective activity of mD8-FAT1 OMVs and EGFRvIII OMVs combination. **(A)** *Analysis of mD8-FAT1 surface expression in B16F10-EGFRvIII cell line*- B16F10-EGFRvIII cells expressing the EGFRvIII human variant were incubated with anti-MD8-FAT1 antibodies and subsequently stained with a fluorescent labeled anti-rabbit antibodies. Antibody binding was followed using flow cytometry analysis. **(B)** *Protection of C57bl6 mice challenged with EGFRvIII-B16F10*. C57bl6 mice were immunized with either mD8-FAT1-OMVs, or EGFRvIII OMVs (20 μg/dose, three doses) or with the combination of mD8-FAT1 OMVs and EGFRvIII OMVs (10 μg/dose each OMV, three doses). Animals were subsequently challenged with 5 × 10^5^ B16F10-EGFRvIII cells and tumor growth was followed over a period of 25 days. The data indicate the average of tumor sizes from each group at the end of the challenge experiment. Means ± SEM are indicated. ***P < 0.001; *P < 0.05.

Several human cancers express EGFRvIII, a variant of EGFR in which a large deletion in its extracellular domain generates a 14 amino acid sequence not found in healthy tissues (22). A vaccine based on EGFRvIII peptide was tested in glioblastoma patients, with promising results even though EGFRvIII-negative tumor cells ultimately escaped vaccine-induced protection (27). We previously demonstrated that OMVs decorated with EGFRvIII peptide elicited specific antibodies which could inhibit the growth of a B16F10 cell line derivative expressing EGFRvIII in syngeneic C57bl6 mice (24). Since EGFRvIII-B16F10 cells, like their progenitor B16F10, express mD8-FAT1 on their surface (Figure 4A), we tested whether the combination of mD8-FAT1-OMVs and EGFRvIII-OMVs could further enhance the anti-tumor activity of EGFRvIII-OMVs immunization in mice challenged with EGFRvIII-B16F10. Mice were immunized three times with either mD8-FAT1-OMVs (20 μg/dose), or EGFRvIII-OMVs (20 μg/dose) or with mD8-FAT1-OMVs + EGFRvIII-OMVs (10 μg each/dose). One week after the third immunization mice were given 10^5^ EGFRvIII-B16F10 cells and tumor growth was followed over a period of 25 days. In line with our previous results (27), at day 25 after challenge, EGFRvIII-OMVs immunization elicited a 70% reduction of tumor growth as compared to immunization with “empty” OMVs. Immunization with mD8-FAT1-OMVs elicited a protection of ~25% (average tumor volume 630 mm^3^ as opposed to 850 mm^3^ in control group). Such protection was lower than what observed in the BALB/c/CT26 model and this is likely due to the fact that B16F10 cells express less mD8-FAT1 than CT26 (Figure 1C). Finally, immunization with the OMVs combination almost totally prevented tumor growth, suggesting that anti-mD8-FAT1 and anti-EGFRvIII antibodies cooperate in inhibiting tumor cell proliferation and in promoting tumor cells killing.

### mD8-FAT1-OMVs synergize with OMVs decorated with cancer T cells epitopes in protecting mice from tumor challenge

Kreiter and co-workers recently reported a list of mutation-derived, CT26-specific CD4+ and CD8+ T cell “neoepitopes” and showed that immunization with RNA vaccines encoding such neoepitopes elicited robust protection in BALB/c mice challenged with CT26 tumor cells (23). We took advantage of these data to address the question as to whether anti-D8-FAT1 immune responses could potentiate the cell-mediated protective activity elicited by T cell neoepitopes.

**Figure 5:**
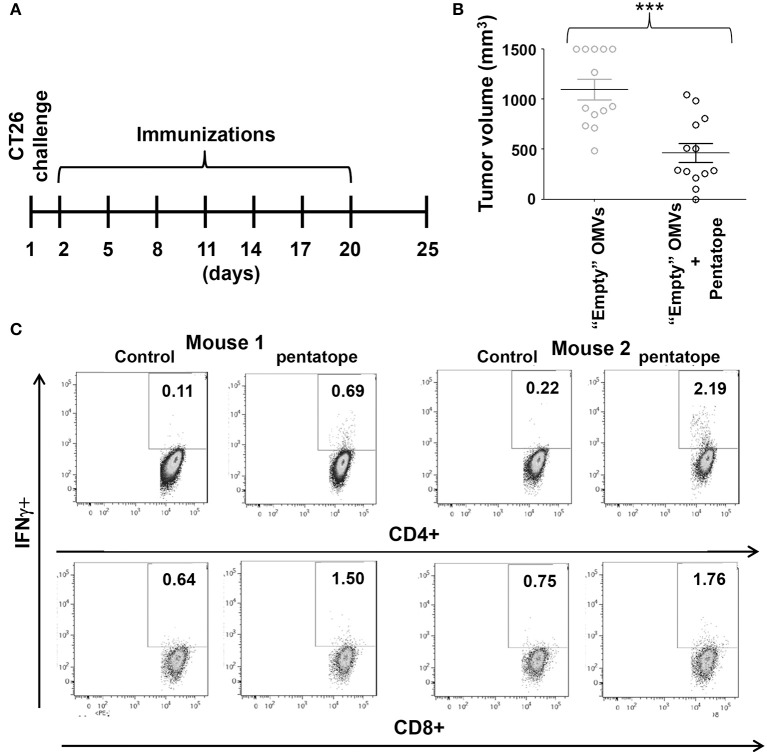
Protective activity of OMVs “absorbed” with synthetic CD4+ T cell epitopes. **(A)** *Schematic representation of tumor protection experiment*. BALB/c mice were challenged with 2 × 10^5^ CT26 cells and the day after were immunized with 20 μg of OMVs mixed with five synthetic peptides (20 μg each) corresponding to CT26-specific CD4+ T cell epitopes (“pentatope”). Immunizations were repeated at a frequency of 3 days and tumor growth was followed over a period of 25 days. **(B)** *Protection of BALB/c mice immunized with “pentatope” OMVs*. The figure reports the tumor volumes measured with a caliper at day 25 after the first immunization (***P < 0.001). Means ± SEM are indicated. **(C)** *Analysis of pentatope-specific CD4*+ *and CD8*+ *T cells in mice immunized with “pentatope”-absorbed OMVs*. BALB/c mice were immunized twice1 week apart with 20 μg of OMVs mixed with five synthetic peptides (20 μg each) corresponding to CT26-specific CD4+ T cell epitopes (“pentatope”). Five days after the second immunization splenocytes were collected and stimulated with either five irrelevant peptides (control) or with the “pentatope” peptide mixture. Induction of IFN-γ expression in CD4+ and CD8+ T cells was analyzed by flow cytometry.

To this aim, we first tested whether OMVs carrying five of the neoepitopes described by Kreiter et al. could protect BALB/c mice from CT26 challenge. Synthetic peptides (20 μg each) corresponding to neoepitopes M03, M20, M26, M27, and M68 (23) were mixed with 20 μg of “empty” OMVs and after challenging mice with 2 × 10^5^ tumor cells the mixture was used to immunize mice every 3 days for a total of 7 injections (Figure 5A). Tumor growth was followed over a period of 25 days. As shown in Figure 5B, immunization with peptides-absorbed OMVs inhibited tumor growth in a statistically significant manner, the average tumor size being 500 ± 94 mm^3^, as opposed to 1.200 ± 103 mm^3^ of control mice. To test whether the observed protection could be at least partially attributable to the elicitation of neoepitope-specific T cells, a group of five naïve mice were immunized twice 1 week apart with peptides-absorbed OMVs and 5 days after the second vaccine dose the frequency of epitope-specific IFN γ-producing T cells was measured in splenocytes stimulated with the peptide mixture. As shown in Figure 5C, which shows the analysis of IFN γ-producing CD4+ and CD8+ T cells from two of the five immunized mice, immunization elicited both epitope-specific CD4+ and CD8+ T cells. The average frequency of CD4+ T cells and CD8+ T cells in the five mice was 0.82 ± 0.29 and 0.48 ± 0.21, respectively.

**Figure 6:**
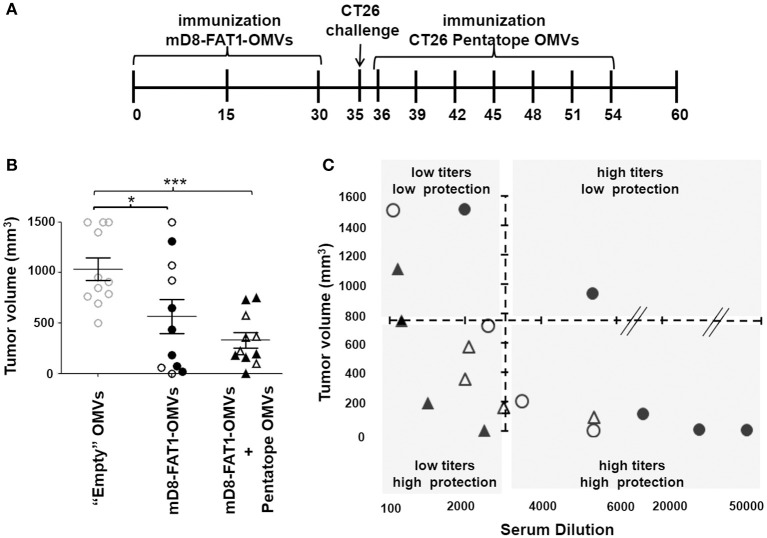
Protective activity of mD8-FAT1-OMVs combined with CD4+ T cell epitopes absorbed to OMVs. **(A)** *Schematic representation of immunization and challenge experiments*. Two groups of BALB/c mice received three doses (2 weeks apart) of either “Empty” OMVs or mD8-FAT1-OMVs (20 μg/dose). Ten days after the last immunization the groups were challenged with 2 × 10^5^ CT26 cells and tumor growth was followed over a period of 25 days. A third group was first immunized with mD8-FAT1-OMVs (20 μg/dose), challenged with 2 × 10^5^ CT26 cells, and finally repeatedly immunized with 20 μg of empty OMVs “absorbed” to the mixture of the five M03, M20, M26, M27, and M68 synthetic peptides (20 μg each). **(B)** *Protection of BALB/c mice immunized with mD8-FAT1-OMVs and with the combination of mD8-FAT1-OMVs with “pentatope” OMVs*. Mice were immunized with mD8-FAT1-OMVs (MBP-mD8-FAT1 OMVs: open circles; FhuD2-mD8-FAT1-OMVs: closed circles) or with the combination of MBP-mD8-FAT1-OMVs + “pentatope” OMVs (open triangles) and FhuD2-mD8-FAT1-OMVs + “pentatope” OMVs (closed triangles) as described in A. The figure reports the tumor volumes measured with a caliper at day 25 after the first immunization (*P < 0.05; ***P < 0.001). Means ± SEM are indicated. **(C)** *Analysis of anti-mD8-FAT1 antibody titers in immunized mice*. At the end of the immunization experiments described in A and B, sera were collected and anti-mD8-FAT1 antibody titers were measured by ELISA. Titers (x axis) are plotted with tumor volumes (y axis). Open and closed circles correspond to sera of mice immunized with MBP-mD8-FAT1-OMVs and FhuD2-mD8-FAT1-OMVs, respectively. Open and closed triangles correspond to sera of mice immunized with MBP-mD8-FAT1-OMVs + “pentatope” OMVs and FhuD2-mD8-FAT1-OMVs + “pentatope” OMVs, respectively.

Having demonstrated that the five neoepitopes described by Kreiter et al. were partially protective when absorbed to OMVs, we next set up an immunization/challenge experiment involving three groups of mice (Figure 6A). Two groups received three doses (2 weeks apart) of either “empty” OMVs or mD8-FAT1-OMVs (20 μg/dose). Ten days after the last immunization the groups were challenged with 2 × 10^5^ CT26 cells and tumor growth was followed over a period of 25 days. A third group was first immunized with mD8-FAT1-OMVs (20 μg/dose), challenged with 2 × 10^5^ CT26 cells and subsequently repeatedly immunized with the mixture of the five M03, M20, M26, M27, and M68 synthetic peptides (20 μg each) absorbed to “empty” OMVs (20 μg). As shown in Figure 6B, the prophylactic mD8-FAT1-OMVs immunization followed by the therapeutic immunization with peptides-absorbed OMVs resulted in a 70% tumor inhibition, the average tumor size at day 25 being 312 ± 76.4 mm^3^.

The protection data obtained in mice immunized with D8-FAT1-OMVs indicate that, although tumor growth was markedly reduced in most of the mice, in few mice immunization was poorly protective (Figures 6B, 3C). By contrast, the tumor size in mice immunized with the combination of D8-FAT1-OMVs and pentatope-absorbed-OMVs was as average not only smaller but also more homogeneous among mice. To explain this difference, we speculated that while in D8-FAT1-OMVs-immunized mice protection was exclusively dependent on anti-D8-FAT1 antibody titers (the higher the titers the better the protection), in mice treated with the OMV combination the antibody titers should have been less critical in protection due to the contribution of cell-mediated immunity. To test this hypothesis, at the end of the experiment described in Figure 6, sera were collected from each mouse and anti-mD8-FAT1 antibody titers were measured in each individual mouse. As shown in Figure 6D, most protected mice (tumor volume <750 mm^3^) immunized with D8-FAT1-OMVs had antibody titers > 1:3.500. By contrast, in mice treated with the OMV combination the same protection was achieved even if anti-D8-FAT1 antibody titers were below 1:3.500.

## Discussion

FAT1 was originally reported as a tumor suppression marker linked to E-cadherin and Wnt/β catenin pathways. Previous evidence from clinical samples showed that, in the presence of wild type FAT1, β-catenin is held at the cell membrane whereas in several tumors, where FAT1 is inactivated by mutation or deleted, an excess of β-catenin is present in the cytoplasm. This results in the inability of the GSK3P/axin/Wtx/Apc complex to completely degrade cytoplasmic β-catenin, allowing active β-catenin to enter the nucleus. Here β-catenin functions as an activator of T-cell factor (TCF) and lymphoid enhancer factor (LEF), leading to a subset of cellular effects involving cellular adhesion, tissue morphogenesis, and tumor development (3).

However, as already pointed out, in some tumors FAT1 is up-regulated, suggesting that its Wnt/β catenin-dependent tumor suppressive mechanism is counterbalanced by a still poorly characterized role as tumor-promoting factor. FAT1 was reported to be overexpressed in breast cancer (28), in melanoma (29) in leukemia (4), and in pancreatic cancer (6). Interestingly, in pancreatic cancer FAT1 was shown to be overexpressed on the surface of cancer cells together with ADAM10 metalloprotease, which mediates FAT1 ectodomain shedding. Although the biological significance of the shed FAT1 ectodomain is unknown, it is possible that it can promote carcinogenesis by disrupting cell junctions and by promoting the up-regulation of metalloproteases, similarly to what has been proposed for the shed ectodomain of E-cadherin (30, 31).

Pileri et al. (7) reported that FAT1 is overexpressed on the surface of most human CRCs and of CRC-derived metastatic hepatocarcinomas. Interestingly, the same authors provided evidence of an ADAM10-dependent FAT1 shedding in HCT15 colon carcinoma cell line, as demonstrated by the accumulation of FAT1 on the cell surface upon siRNA-mediated silencing of ADAM10 mRNA.

While for many tumors the opposite role of FAT1 as tumor suppressor and tumor promoter is at present difficult to reconcile, in the case of CRC there might be a mechanistic explanation. In virtually all colon carcinomas β-catenin degradation is hampered by defects in the Apc subunit of the GSK3P/axin/Wtx/Apc complex (32). Therefore, even if FAT1 overexpression should reduce the concentration of free cytoplasmic β-catenin, the inability to degrade it should allow enough β-catenin to reach the nucleus and activate genes involved in tumorigenesis. At the same time, the abundancy of surface FAT1 and its shed extracellular ectodomain should promote carcinogenesis.

In this work we wanted to investigate whether FAT1-based cancer vaccines could be potentially exploited in CRC immunotherapy. The rationale behind this work stems from a number of experimental observations. First, a monoclonal antibody (mAb198.3) specific for a conserved amino acid sequence in D8 and D12 FAT1 domains could bind the surface of over 90% of CRCs with affinities in the low nM range. Second, cancer cell recognition by mAb198.3 appeared rather specific. IHC analysis of 33 normal human tissues showed a limited recognition of any of the tissues by mAb198.3 and, when present, the staining was confined to the intracellular compartment. Third, when used in xenograft mouse models with HCT15 and HT29 cell lines, mAb198.3 passive immunization could reduce tumor growth in a statistically significant manner. However, for a FAT1-based vaccine to be effective and safe a fundamental requisite is the absence of FAT1, and in particular of D8-FAT1 epitope, surface expression in healthy tissues. Only under these circumstances the central clonal deletion of FAT1-spcific naïve B cells is avoided together with the risk of inducing immune responses which could be detrimental to immunized patients. While the effectiveness and safety of FAT1 vaccine can ultimately be demonstrated only in humans, to move to the clinics robust preclinical and safety data are required.

Starting from the assumption that mice and humans share a similar FAT1 expression profile, we decided to test FAT1-based vaccines in an immune competent mouse model. First we analyzed whether the mouse homolog of human FAT1 was expressed in some of the cell lines most frequently utilized in mice. Indeed, as judged by quantitative RT PCR analysis of FAT1 mRNA, we found that FAT1 is overexpressed in a number of cancer cell lines, including B16F10 and, particularly, CT26. This was encouraging, considering that CT26 cell line derives from a spontaneous colon cancer of BALB/c mice.

We next aligned the sequences of hFAT1 and mFAT1 and we selected the mFAT1 25 amino acids (mD8-FAT1) corresponding to the human epitope (hD8-FAT1) recognized by mAb198.3. Interestingly, mD8-FAT1 differs in 4 out of 25 amino acids from hD8-FAT1 and this difference is sufficient to abrogate the binding of mAb198.3 to mD8-FAT1.

We then asked the question whether mD8-FAT1 epitope could induce FAT1-specific antibody responses in mice. This was a critical question since, as said above, if mFAT1 were sufficiently expressed on the surface of normal tissues, the administration of mD8-FAT1 containing vaccines should be poorly immunogenic and/or potentially harmful. Interestingly, the mD8-FAT1 immunization elicited high titers of specific antibodies and although we did not carry out a pathological/histopathological analysis of immunized mice, the animals showed no severe sign of malaise and/or alteration of physiological functions throughout the experiment. This result is in line with the immunohistochemistry data, which indicate the absence of surface expression of hD8-FAT1 domain on normal human tissues (7) and suggests that in mice FAT1 has a topological organization and an expression profile similar to what observed in humans.

Next, we investigated whether the immune response induced by mD8-FAT1 vaccination was potent enough to inhibit tumor growth in BALB/c and C57bl6 mice challenged after vaccination with CT26 and B16F10 cells, respectively. Our data indicate that mD8-FAT1 immunization did reduce the kinetics of tumor development in both animal models, even though it was not capable of fully abrogating tumor formation. Protection was more pronounced in the BALB/c-CT26 model and this is likely due to the fact that FAT1 expression is fourfold higher in CT26 than in B16F10 (Figure 1). Interestingly, when we analyzed the cell population in tumors from immunized BALB/c mice we found an increase in infiltrating CD4+ and CD8+ T cells and a concomitant decrease in Treg and MDSCs with respect to tumors from mock immunized mice (Figure 3). This is in line with one of the expected mechanisms of action of anti-tumor antibodies according to which ADCC-mediated killing of cancer cells creates an inflammatory environment and favors the infiltration of effector T cells specific for cancer epitopes released by dead cells.

While ADCC is likely to play an important role in the observed anti-tumor activity, other possible mechanisms can be involved including a direct cell killing or growth inhibition mediated by antibody binding to target cells. To investigate the involvement of this latter mechanism we carried out *in vitro* experiments in which CT26 cells were incubated for 72 h with different concentrations of affinity-purified anti-mD8-FAT1 polyclonal antibodies and we followed cell proliferation using the MTT assay (Promega). As shown in Supplementary Figure 1, the addition of affinity-purified anti-mD8-FAT1 polyclonal antibodies partially inhibited cell growth (~20%) in a dose-dependent manner. No growth inhibition was observed when the cells were incubated with pre-immune serum, and with similar concentrations of purified polyclonal antibodies against an unrelated peptide.

Finally, we investigated whether the tumor inhibiting activity elicited by mD8-FAT1 immunization could be potentiated by the combination with other tumor-specific antigens. Targeting single antigens would hardly be effective in cancer immunotherapy and therefore the capacity of antigens to synergize with others would be an important prerequisite in the final selection of the proper vaccine combinations. Our data indicate that when mD8-FAT1 is combined with other B and T cells protective epitopes the anti-tumor immune response is potentiated. In particular, the immunization with mD8-FAT1 combined with EGFRvIII, a B cell epitope expressed in a variety of tumors in which the EGF receptor undergoes the deletion of its ectodomain (22), almost fully abrogated tumor development in C57bl6 mice challenged with B16F10-EGFRvIII, a cell line expressing both mD8-FAT1 and EGFRvIII. Our data not only point to the effectiveness of antibody-mediated immunotherapies targeting more than one tumor-specific B cell antigen/epitope but also suggest that the combination of D8-FAT1 and EGFRvIII might find practical applications in CRC patients since EGFRvIII expression has been described in at least a subset of human colorectal cancers (33).

In this work we also show the additive protective activity of mD8-FAT1 when combined with cancer-specific T cell epitopes. In the last few years CD4+/CD8+ T cell neoepitopes originated from cancer mutations are emerging as key targets for cancer immunotherapy (34). This has been proved in the clinical settings for both adoptive cell transfer therapy (ACT) (35) and cancer vaccines (36). In the case of cancer vaccines, the combination of more than one cancer-specific T cell neoepitope was critical for the effectiveness of the vaccines (20, 21). As already pointed out in a recently published work from our laboratories (27), the fact that the potency of multi-T cell epitopes vaccines can be further potentiated by the addition of protective B cell epitopes expand the potential of future cancer vaccines.

It has to be pointed out that our data on B and T cell epitope combinations should be taken as a “proof-of-concept” study that needs further optimization. According to the protocol utilized in this work, before the challenge with CT26 cells, the animals are immunized first with mD8-FAT1 to elicit sufficiently high FAT1-specific antibody titers. The challenge is then followed by repeated immunizations with T cell epitopes absorbed to OMVs. The reason why we followed this protocol in our “proof-of-concept” experiments is because previously described work with Rindopepimut, an anti-glioblastoma EGFRvIII peptide vaccine, highlighted the importance of implanting mice with high doses of cancer cells only in the presence of sufficiently high antibody titers (37, 38). It would be interesting to follow protection when B and T cell epitopes are combined and given to mice simultaneously after tumor challenge. Preliminary data seem to indicate that such schedule is not as effective as the one here described but before drawing any conclusion further experiments involving different antigen dosages, formulations, and timing should be carried out.

A last but important comment deserves the adjuvant/formulation used in this study. Several adjuvants/delivery systems have been proposed for cancer vaccines, including DNA and RNA encoding cancer antigens/epitopes, synthetic peptides combined with hiltonol, and viral vectors expressing cancer antigens (36). We tend to believe that OMVs are a valid and promising alternative. As already pointed out, OMVs have a few interesting properties. They carry many Microbe-Associated Molecular Patterns (MAMPs), which can work synergistically, thus providing a strong built-in adjuvanticity to OMVs (11, 12). Furthermore, OMVs can be easily decorated with foreign antigens/epitopes by manipulating the OMV-producing strains (13–17). Finally, OMVs can be rapidly and easily purified from bacterial culture supernatant using either detergent treatment of bacterial cells (18), or hyper-vesiculating strains (19). We had previously shown that OMVs engineered with EGFRvIII peptide and a CD4+ T cell epitope fully protected C57bl6 mice from the challenge of B16F10-EGFRvIII cell line and that protection strongly correlated with the elicitation of both humoral and cell-mediated immunity. The data described in this work further support our motivation to exploit OMVs in cancer immunotherapy.

## Materials and methods

### Bacterial strains, cell line, and mice

*E. coli* HK100 strain was used for cloning experiments with the PIPE method. *E. coli* BL21(DE3)Δ*ompA* strain used for OMVs production was previously described (16). CT26 and B16F10 were obtained from ATCC (Manassas, VA, USA) and cultured under recommended conditions. B16F10 melanoma cell line that stably expresses human EGFRvIII variant was generously provided by Prof. Sampson (Department of Neurosurgery of the Duke University, Duhram, NC). Cells were tested for mycoplasma before animal injection.

BALB/c and C57bl6 female 4 weeks old mice were purchased from Charles River Laboratories and kept and treated in accordance with the Italian policies on animal research at the Toscana Life Sciences animal facility (Siena, IT).

### Construction of plasmids

Three copies of mD8-FAT1 were fused to the C-terminus of the *S. aureus* FhuD2 lipoprotein. D8-mFAT1 minigene was constructed, taking into consideration BL21 *E. coli* codon usage, by assembling six complementary oligonucleotides, the sequence of which is reported in Table 1, and the assembled DNA fragment was amplified with primers fat1ms-FhUD2 F/ fat1ms-FhUD2 R primer (Table 1). These primers were designed to generate extremities complementary to the pET-FhuD2 plasmid. This vector, which carries the *fhuD2* gene fused to the *lpp* leader sequence (39) was linearized by PCR amplification using the divergent primers nohis flag F/ FhuD2-V-R, according to the PIPE method (40). Finally, the PCR products were mixed together and used to transform HK-100 competent cells, obtaining plasmids pET_FhuD2-mD8-FAT1-3x plasmid.

**Table 1:** Oligonucleotide primers used for mFAT1 minigene preparation.

**NAME**	**SEQUENCE**
mFa-F1	ATCCAAGTGGAGGCGACCGATAAAGACCTGGGTCCGTCGGGGCATGTG
mFa-R1	AACCTGAATTTCGGTGTCGGTCAGGATGGCATACGTCACATGCCCCGACGG
mFa-F2	ACCGAAATTCAGGTTGAAGCCACCGACAAAGACTTAGGCCCGAGTGGTCAC
mFa-R2	CTGAATTTCAGTATCGGTGAGAATCGCGTAGGTCACGTGACCACTCGGGCC
mFa-F3	GATACTGAAATTCAGGTTGAAGCTACCGATAAAGATTTGGGCCCGAGTGGT
mFa-R3	TTCAGTATCCGTGAGGATCGCATAGGTTACATGACCACTCGGGCCCAA
**pET_FhuD2-D8-mFAT1-3x, pET_MBP-D8-mFAT1-3x**	
nohis flag	CATCACCATCACCATCACGATTACA
fat1ms- FhUD2 F	TAATTAAAGCTGCAAAAATCCAAGTGGAGGCGACCGA
fat1ms- FhUD2 R	GATGGTGATGGTGATGTCATTCAGTATCCGTGAGGATCG
FhUD2-V-R	TTTTGCAGCTTTAATTAATTTTTC
MBPmFa-F	CGCGCAGACTCGTATCACCAAGATCCAAGTGGAGGCG
MBPmFa-R	TCGTGATGGTGATGGTGATGTTATTCAGTATCCGTGAG
pET21-MBPF	CATCACCATCACCATCACGATTAC
pET21-MBPR	CTTGGTGATACGAGTCTGCGCGTC

Similarly, to express mD8-FAT1 peptide in the lumen of OMVs, the Maltose binding protein (MBP) was used as a carrier and the FAT1 minigene was cloned as an in frame fusion to the 3′ end of the MBP gene. For this purpose, pET-MBP plasmid (41) was used as template for a PCR reaction carried out using primers pET21-MBPF and pET21-MBPR (see Table 1) to generate a linear fragment. Then, the linear fragment was ligated to mD8-FAT1 3x gene, which was assembled as previously described and subsequently amplified with primers MBPmFa-F and MBPmFa-R. The DNA mixture was used to transform HK-100 competent cells and clones carrying pET_MBP-mD8-FAT1 plasmid were selected on LB agar plates supplemented with 100 μg/ml of Ampicillin. The correctness of the mD8-FAT1 fusions from one of the Ampicillin resistant clones was verified by DNA sequencing.

The construction of pET-Nm-fHbp-vIII plasmid expressing the *Neisseria meningitidis* FHbp fused to three repeated copies of EGFRvIII peptide was previously described (24).

### Synthetic peptides and antibodies

The mD8-FAT1 peptide IQVEATDKDLGPSGHVTYAILTDTE unconjugated and conjugated to KLH protein was purchased from Proteogenix in lyophilic form and solubilized in PBS at the final concentration of 1 mg/ml. Polyclonal antibodies against mD8-FAT1 peptide were obtained from Proteogenix by immunizing rabbits with KLH-conjugated IQVEATDKDLGPSGHVTYAILTDTE peptide.

The synthetic peptides M03 (DKPLRRNNSYTSYIMAICGMPLDSFRA), M20 (PLLPFYPPDEALEIGLELNSSALPPTE), M26 (VILPQAPSGPSYATYLQPAQAQMLTPP), M27 (EHIHRAGGLFVADAIQVGFGRIGKHFW) and M68 (VTSIPSVSNALNWKEFSFIQSTLGYVA) were purchased from GeneScript in lyophilic form and solubilized in milliQ water at final concentration of 5 mg/ml.

### Preparation of bacterial total lysates and OMVs

Plasmids containing the genes of interest were used to transform *E. coli* BL21(DE3)Δ*ompA* strain. Recombinant clones were grown in 200 ml LB medium (starting OD_600_ = 0.05) and, when the cultures reached an OD_600_ value of 0.5, protein expression was induced by addition of 1 mM IPTG. After 2 h, OMVs were collected from culture supernatants by filtration through a 0.22 μm pore size filter (Millipore) followed by high-speed centrifugation (200,000 × g for 2 h). Pellets containing OMVs were finally re-suspended in PBS. Total bacterial lysates were prepared by suspending bacterial cells from 1 ml cultures (centrifuged at 13,000 × g for 5 min) in sodium dodecyl sulfate-polyacrylamide gel electrophoresis (SDS-PAGE) Laemmli buffer and heated at 100°C for 5 min. Proteins were separated by 4–12% or 10% SDS-PAGE (Invitrogen), run in MES buffer (Invitrogen) and finally stained with Coomassie Blue.

### Flow cytometry analysis

Twenty milliliter of LB medium supplemented with 100 μg/ml Ampicillin were inoculated at OD_600_ = 0.05 with an overnight culture of BL21Δ*ompA*(pET_Empty), BL21Δ*ompA*(pET-pET_MBP-mD8-FAT1), and BL21Δ*ompA*(pET-pET_FhuD2 mD8-FAT1) strains. The cultures were then grown and IPTG-induced as described above. BL21Δ*ompA*(pET_Empty) strain was used as negative control. Bacterial cells from 1 ml were harvested by centrifugation at 10,000 × g for 5 min at 4°C and re-suspended with 1% BSA in PBS to obtain a cell density of 2 × 10^7^ CFUs/ml. 50 μl were then dispensed in a round bottom 96 well plate. Anti-mD8-FAT1 peptide rabbit antibodies were added at a concentration of 5 μg/ml and incubated 1 h on ice. After three washes with 1% BSA in PBS, 20 μl of FITC labeled anti-rabbit secondary antibodies (1:200 dilution) (Life Technologies) were added and incubated 30 min on ice. Each well was then washed twice with 200 μl 1% BSA in PBS and plates were centrifuged at 4,000 × g for 5 min. Samples were then re-suspended in 2% formaldehyde solution, incubated 15 min at RT and centrifuged again at 4,000 × g for 5 min. Finally, samples were re-suspended in 200 μl of PBS and data were acquired by using BD FACS Canto II cell analyzer (BD) and analyzed by FlowJo software.

### Triton X-114 protein separation from OMVs

One hundred micrograms of OMVs (10–15 μl) were diluted in 450 μl of PBS, then ice cold 10% TritonX-114 was added to 1% final concentration and the OMV-containing solution was incubated at 4°C for 1 h under shaking. The solution was then heated at 37°C for 10 min and the aqueous phase was separated from the detergent by centrifugation at 13,000 × g for 10 min. Proteins in both phases were then precipitated by standard chloroform/methanol procedure, separated by SDS-PAGE electrophoresis and stained with Coomassie blue.

### Vaccine immunogenicity and tumor challenge

#### OMV immunizations

BALB/c mice were vaccinated on day 0, 14, and 28 with 20 μg of either “empty” OMVs [derived from BL21Δ*ompA* (pET21_Empty)] or MBP-mD8-FAT1-OMVs strain supplemented with Alum, or FhuD2-mD8-FAT1 strain. At day 35, 2 × 10^5^ CT26 cells were subcutaneously (s.c.) injected in each animal and tumor growth was measured with a caliper every 3 days over a period of 30 days. For ethical reasons, mice were euthanized when tumors reached a size of 1,500 mm^3^.

#### Immunization with MBP-mD8-Fat1-OMVs combined with Nm-fHbp-vIII OMVs

C57bl6 mice (8 mice/group) were vaccinated on day 0, 14, and 28 with 20 μg “empty” OMVs, 20 μg MBP-mD8-FAT1-OMVs, 20 μg Nm-fHbp-vIII-OMVs and 10 μg of MBP-mD8-FAT1-OMVs + 10 μg *Nm-*fHbp-vIII-OMVs supplemented with Alum. At day 35, 10^5^ B16F10-EGFRvIII cells were s.c. injected in each animal and tumor growth was followed as described above.

#### Immunization with mD8-FAT1-OMVs combined with CT26 neo-epitopes

BALB/c mice (six mice/group) were i.p. vaccinated on day 0, 14, and 28 with 20 μg of either “empty” OMVs (Group1) or mD8-FAT1-OMVs (group 2 and 3). At day 35, 2 × 10^5^ CT26 cells were subcutaneously (s.c.) injected in each animal as previously described. The following day mice of group 3 were i.p. immunized with 20 μg of “empty” OMVs supplemented with 20 μg each of the five CT26 peptides (“pentatope”). Immunizations were repeated every 3 days for seven times and tumor growth was followed as described above.

#### Analysis of anti-mD8-FAT1 antibodies in immunized animals

Anti-mD8-FAT1 antibodies were measured by ELISA. Amino plates (Thermo Fisher) were coated with synthetic mD8-FAT1 peptide (0.5 μg/well) and incubated overnight at 4°C. The day after, plates were saturated with a solution of 1% BSA in PBS (200 μl per well) for 1 hr at 37°C. Mice sera were threefold serially diluted in PBS supplemented with 0.05% tween (PBST) and 0.1 % BSA. After 3 washes with PBST, 100 μl of each serum dilution were dispensed in plate wells. As positive control, Anti-mD8-FAT1 rabbit serum from animals immunized with KLH-conjugated IQVEATDKDLGPSGHVTYAILTDTE peptide was used. After 2 hr incubation at 37°C, wells were washed three times with PBST and then incubated 30 min at 37°C with mouse anti-rabbit alkaline phosphatase-conjugate antibodies at a final dilution of 1: 2,000. After 3 washes with PBST, 100 μl of Alkaline Phosphatase substrate (Sigma Aldrich) were added to each well and plates were maintained at room temperature in the dark for 30 min. Finally, absorbance was read at 405 nm using the M2 Spectramax Reader plate instrument.

#### T cell analysis

At the end of the tumor challenge studies described above (30 days from tumor cell administration) mice were sacrificed and spleens collected in 5 ml DMEM high glucose (GIBCO). Spleens were then homogenized and splenocytes filtered using a Cell Strainer 70 μm. After centrifugation at 400 × g for 7 min, splenocytes were suspended in PBS and aliquoted in a 96 well plate at a concentration of 1 × 10^6^ cells per well. Cells were stimulated with 10 mg/ml of an unrelated peptide (negative control), or 10 mg/ml each of a mix of the five peptides that make up the “pentatope” (M03, M20; M26, M27, and M68 peptides). As positive control, cells were stimulated with phorbol 12-myristate 13-acetate (PMA, 0.5 mg/ml) and Ionomycin (1 mg/ml). After 2 h of stimulation at room temperature, Brefeldin A [Beckton Dickenson (BD)] was added to each well and cells incubated for 4 h at 37°C. After 2 washes with PBS, NearIRDead cell staining reaction mixture (Thermo Fisher) was incubated with the splenocytes for 20 min at room temperature in the dark. After two washes with PBS and permeabilization and fixing with Cytofix/Cytoperm (BD) using the manufacturer's protocol, splenocytes were stained with a mix of the following fluorescent-labeled antibodies: Anti CD3-APC (BioLegend), Anti-CD4-BV510 (BioLegend), anti-CD8-PECF594 (BD) and IFN–γ-BV785 (BioLegend). Samples were analyzed on a BD LSRII FACS using FlowJo software. Graphs were processed with Prism 5.0 software (Graphpad). Statistical analysis and differences in means between two groups were compared by unpaired, two-tailed Student's *t*-test (n.s.: *P* > 0.05, ^*^P < 0.05, ^**^P < 0.01, ^***^P < 0.001).

#### Analysis of tumor infiltrating lymphocytes

Tumor infiltrating lymphocytes were isolated from subcutaneous CT26 tumors taken from sacrificed mice. At least two tumors per group were collected and minced into pieces of 1–2 mm of diameter using a sterile scalpel. Tumor samples were then transferred into a 15 ml tubes containing 5 ml of collagenase solution (Collagenase Type 3 200 U\ml, Collagenase Type 4 200 U\ml) diluted in HBSS with 3 mM CaCl_2_ and incubated under agitation for 2 h at 37°C. The resulting cell suspensions were filtered through a Cell Strainer 70 μm, washed twice with PBS and 1 × 10^6^ cells were dispensed in a 96 well plate. Then cells were incubated with NearIRDead cell staining Kit (Thermo Fisher) 20 min on ice in the dark. After two washes with PBS, samples were stained with the following mixture of fluorescent-labeled antibodies (BD): anti GR1 (BV605), anti–CD11b-BV480, anti-CD45-BV786, anti-CD4-PE, and anti-CD8-PECF594. The samples were then incubated 1 h at RT. After 2 washes with PBS, Cytofix/Cytoperm (BD) was added to each well and incubated 20 min on ice in the dark. After 2 washes with PBS, cells were stained with anti-Foxp3-A488 (BD) antibodies diluted in Permwash 1X buffer 20 min at RT in the dark. Finally, samples were washed 2 times with 1% BSA in PBS and analyzed on a BD LSR II FACS as described above.

### RNA extraction and qRT-PCR analysis

RNA extraction from cell lines was performed using the RNeasy mini kit (QIAGEN) and 500 ng of it were reverse transcribed using Superscript III Reverse Transcriptase (Life Technologies) with oligo dT. Triplicate cDNA samples from each cell line (equal to 50 ng RNA/sample) were subjected to qRT-PCR to assess the relative FAT1 (Quantitect® Primer Assay for mouse FAT1, QIAGEN) transcript levels using the Quantitect® SYBR Green PCR kit (QIAGEN). MAPK, actin (Quantitect® Primer Assay for Human actin or MAPK, QIAGEN), were used as an internal normalization controls, respectively. Data were analyzed with the One-Step Plus qRT-PCR equipment (Applied Biosystems).

### Cytotoxicity assay

5 × 10^4^ CT26 cells were plated in triplicate in a 96 wells plate in RPMI medium + 10% FBS (GIBCO). Cells were incubated for 72 h at 37°C with three different concentrations (10 μg/ml, 5 μg/ml and 1 μg/ml) of affinity-purified rabbit anti-mD8-FAT1 polyclonal antibodies. As controls CT26 cells were incubated with PBS, pre-immune serum from the same rabbit used for D8-FAT1 immunization (1:1000 final dilution), or with three concentrations of rabbit polyclonal antibodies against an unrelated peptide. Cell proliferation was followed using the CellTiter 96 Non-Radioactive Cell Proliferation Assay (Promega) according to the manufacturer's protocol. Finally, absorbance was read at 750 nm using the M2 Spectramax Reader plate instrument.

## Ethics statement

Mice were monitored twice per day to evaluate early signs of pain and distress, such as respiration rate, posture, and loss of weight (more than 20%) according to humane end points. Animals showing such conditions were anesthetized and subsequently sacrificed in accordance with experimental protocols, which were reviewed and approved by the Animal Ethical Committee of Toscana Life Sciences Foundation and by the Italian Ministry of Health.

## Author contributions

AlG and MP cloning, expression, purification OMVs, challenge studies, flow cytometry, and manuscript revision. SV mouse models. ST and CS, flow cytometry. LaF, CI, RC, EC, and SS cloning and OMV preparation, antigen compartmentalization in OMVs. MT and IZ flow cytometry, T cell analysis and tumor challenge. LuF, LG, EK, SI, and AsG WB, protein analysis and ELISA. GG experimental design, project coordination, and manuscript preparation.

### Conflict of interest statement

The authors declare that the research was conducted in the absence of any commercial or financial relationships that could be construed as a potential conflict of interest.
