# Supplementary figures and images for: Vaccination With a FAT1-Derived B Cell Epitope Combined With Tumor-Specific B and T Cell Epitopes Elicits Additive Protection in Cancer Mouse Models
Source: Front Oncol. 2018 Oct 26;8:481. doi: 10.3389/fonc.2018.00481 (PMC6212586; doi:10.3389/fonc.2018.00481)

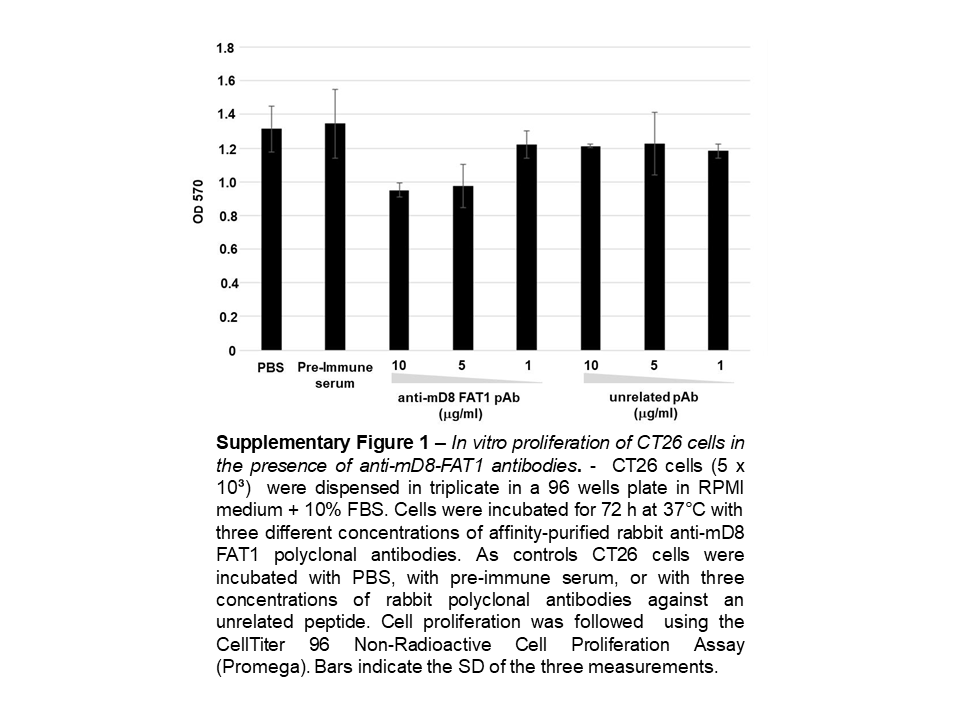

Supplement: Supplementary file 1 [file Image_1.TIF]
